# NETs unleashed: neutrophil extracellular traps boost chemotherapy against colorectal cancer

**DOI:** 10.1172/JCI178344

**Published:** 2024-03-01

**Authors:** Alexandra Mousset, Jean Albrengues

**Affiliations:** University Côte d’Azur, CNRS UMR7284, INSERM U1081, Institute for Research on Cancer and Aging, Nice (IRCAN), Nice, France.

## Abstract

Chemotherapy, which primarily acts on cancer cells, can influence the tumor microenvironment and the recruitment and behavior of stromal cells. In this issue of the *JCI*, Li et al. explored the potent anticancer effect of the combination of a glutaminase inhibitor (CB-839) and 5-FU against *PIK3CA*-mutant colorectal cancer tumors. This chemotherapy treatment strongly induced the recruitment of neutrophils that formed neutrophil extracellular traps in cancer, which actively killed cancer cells by inducing apoptosis. This study substantially advances our understanding of the multifaceted role of neutrophils and NETs in the outcome of anticancer treatment.

## CB-839 and 5-FU combination shrinks PIK3CA-mutant colorectal cancer tumors

The tumor microenvironment (TME) is now widely recognized as a crucial factor influencing the efficacy of chemotherapy. When chemotherapy is effective, it induces cell damage and death, which substantially impacts the TME, including the recruitment and behavior of inflammatory cells. High neutrophil infiltration is commonly linked to a poor response to chemotherapy in various human cancer types (1). However, exceptions have been observed in colorectal, gastric, and high-grade ovarian cancers, where increased neutrophil levels are associated with a better response to chemotherapy (2, 3). In this issue of the *JCI*, Li and colleagues focused on the impact of chemotherapy on colorectal cancer (CRC), specifically those cases with *PIK3CA* mutations, which account for 30% of CRC cases (4). The authors had previously demonstrated that *PIK3CA* mutations make CRC more dependent on glutamine and therefore the combination of a glutaminase inhibitor (CB-839) and 5-FU leads to notable regression of *PIK3CA*-mutant CRC in xenograft nude mouse models (5, 6). Building on this foundation, Li and colleagues aimed to investigate the cellular and molecular mechanisms underlying the potent anticancer effect of this drug combination, referred to as the combo drug (4).

## Neutrophil extracellular traps modulate CB-839/5-FU combo antitumor effects

**Figure 1:**
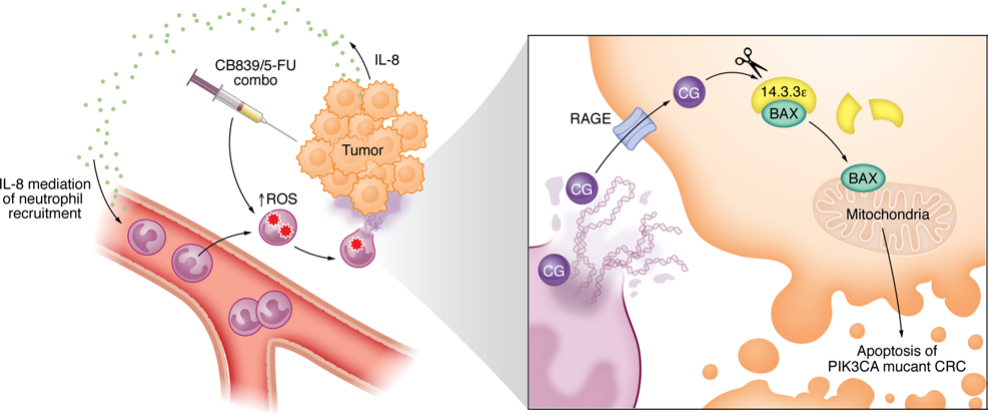
NETs induced by chemotherapy inhibit CRC tumor growth. Li and colleagues (4) present a mechanism by which NETs formed in response to chemotherapy induce PIKCA-mutant CRC cell apoptosis. Combination treatment of a CB839 and 5-FU upregulates IL-8 secretion by cancer cells, resulting in neutrophil recruitment in tumors. In addition, the CB-839/5-FU combination treatment induces ROS accumulation in neutrophils, which results in NET formation. NETs contain CG, which can enter in cancer cells via the cell surface protein RAGE. Once internalized, CG cleaves 14-3-3^ε^, which induces BAX mitochondrial translocation, triggers apoptosis, and results in tumor regression.

Focusing on innate immune cells, the authors showed that while neither macrophage nor NK cell depletion influenced chemotherapy response, neutrophil depletion blocked the efficacy of the combo drug on multiple CRC xenograft tumor models. Indeed, following the combo drug treatment, neutrophils were massively recruited into tumors and formed neutrophil extracellular traps (NETs). NETs are scaffolds of DNA with several cytotoxic enzymes and proteases that are released by neutrophils, and NETs have recently emerged as strong modulator of anticancer treatment (7). Even though NETs were previously described to protect cancer cells from chemotherapy and radiotherapy (8–10), Li, et al. established that NET digestion with DNase I strongly inhibited the combo drug efficacy in their models. The authors next investigated the molecular mechanisms involved. Interestingly, *PIK3CA*-WT tumors had much less infiltration of NET-forming neutrophils following the combo drug treatment. Taking advantages of these differences, the authors performed RNA-Seq on *PIK3CA* WT and mutant tumors and identified an enrichment of the neutrophil chemoattractant IL-8 in *PIK3CA*-mutant cancer cells. Even though IL-8 is specific to humans, it can also attract mouse neutrophils (11). Accordingly, KO of IL-8 in different CRC cell lines attenuated neutrophil recruitment and NET formation and counteracted the combo drug efficacy (Figure 1). In addition, the authors were able to show that the induction of IL-8 transcription following treatment with the combo drug was mediated by the transcriptional factor Nuclear factor (erythroid-derived 2)-like 2 (NRF2), which bound to the promoter region of the IL-8 gene. The authors also identified that the combo drug directly induced NETs, which was dependent on ROS production (Figure 1).

## NET-associated Cathepsin G induces cancer cell apoptosis

To gain insight into how NETs participate in the shrinkage of CRC following the combo drug treatment, the authors turned to in vitro experiments and demonstrated that NETs, induced by the combo drug, directly induced the apoptosis of cancer cells. Similarly, the combo drug induced apoptosis in vivo, a process that was diminished following neutrophil depletion or DNase I digestion of NETs. The authors thus proposed that NETs may directly contribute to CRC cell death by inducing apoptosis. NETs are associated with granular proteins, including several proteases like Neutrophil elastase, Matrix MetalloProteinase 9, and Cathepsin G (CG) (12, 13). The authors first demonstrated that free recombinant CG could promote CRC apoptosis and, conversely, the inhibition of CG attenuated NET-induced apoptosis in vitro. Consistently, the inhibition of CG counteracted the efficacy of the combo drug and apoptosis in vivo. Although CG alone was capable of inducing cancer cell apoptosis, suggesting that the NET-DNA scaffold may not be necessary, treatment with DNase I blocked combo-induced apoptosis. The authors hypothesized that the decondensed DNA in NETs could anchor CG within tumors. Supporting this concept, Western blot analysis of tumors treated with the combo drug showed a reduction in CG following DNase I treatment. Previous studies have described that the cell surface protein receptor for advanced glycation end products (RAGE) mediates neutrophil-derived CG cytotoxicity (14). Using KO techniques in vitro, the authors demonstrated that CG entered cancer cells through RAGE. Accordingly, RAGE-KO cells were less sensitive to the combo drug than control cells. Notably, once inside cancer cells, CG cleaved 14-3-3^ε^ proteins, which normally bind and sequester Bcl-2–associated X protein (Bax) from mitochondria to prevent apoptosis (15). Accordingly, 14-3-3^ε^ cleavage and the subsequent apoptosis were blocked by a CG inhibitor and DNase I (Figure 1).

## Relevance to human CRC

Taking advantage of biopsies from patients enrolled in a phase II clinical trial testing a combination of CB-839 with capecitabine (an oral prodrug of 5-FU), Li and colleagues assessed the clinical relevance of their multiple preclinical models. Although no objective response was observed in patients treated with the combo drug, increased levels of NETs in posttreatment tumor biopsies (characterized by elevated cit-H3 levels) were associated with longer progression-free survival.

## Clinical and research implications

Neutrophils play a dual role in cancer, showing pro- and antitumor activities. This duality extends to cancer treatments like radiotherapy, immunotherapy, and now chemotherapy (7). Previously recognized for their role in countering chemotherapy (8, 10), neutrophils, as revealed by Li et al., can enhance chemotherapy effectiveness through the release of NETs.

The dual role of neutrophils in chemotherapy outcomes raises new questions. While another study recently described that chemotherapy-induced IL-1β triggers NETs, which, in turn, promote chemoresistance against breast cancer lung metastasis (8), Li et al., demonstrate that the combo drug directly induces NETs to enhance chemotherapy efficacy against *PI3KCA*-mutated CRC. These two processes not only differ in triggering mechanisms but also in downstream effects. The first associates NETs with TGF-β activation, leading to epithelial to mesenchymal transition (EMT) and chemoresistance, while the second highlights the involvement of NETs in the direct promotion of cancer cell apoptosis via NET-associated CG and its effects on 14-3-3^ε^ protein degradation. In addition, different types of chemotherapy that were used in these study (e.g., AC chemotherapy for breast cancer versus the combo drug for CRC) might be responsible for the differences observed. These findings also challenge the belief that NETs primarily exert antiapoptotic effects (16), prompting critical questions about contextual determinants for NET behavior in cancer. Differences between “chemoresistant NETs” and “proapoptotic NETs” may rely on various factors, including NET-inducing agents, NET types, or targeted cells. Li et al. suggest that NETs induced by the combo drug differ from PMA-induced NETs, indicating the existence of distinct NET subtypes or compositions. Considering this heterogeneity, targeting specific NET types could provide a nuanced therapeutic approach. The mutational landscape of cancer may also regulate neutrophils’ different roles in chemotherapy responses. The combo drug induces NETs specifically in *PI3KCA*-mutated CRC, suggesting a mutational influence. However, assessing whether these NETs enhance apoptosis of WT cells and exploring the regulation of the cell surface receptor RAGE by *PI3KCA* mutation remains unexplored.

The authors propose that the binding of NETs to CRC cells is crucial for preventing the washout of CG in the TME. Interestingly, Li and colleagues reveal that coiled-coil domain containing protein 25 (CCDC25), a previously described NET receptor (17), is not involved in NET-induced apoptosis, suggesting the involvement of possible alternative binding mechanisms. Indeed, unraveling the mechanisms by which NETs bind to cancer cells could lead to a more specific understanding of the cancer cell response to NETs. Notably, recent findings indicate that NETs can trap doxorubicin, therefore limiting chemotherapy efficacy (10) and that NETs can shield cancer cells from cytotoxicity mediated by CD8^+^T cell natural killer cells, limiting immunotherapy efficacy (18). In addition, Li and authors show that CG from NETs enters cancer cells to induce apoptosis. It would be interesting to assess whether DNA fragments from the NETs also enter cancer cells with CG, and whether this NET-DNA complex could activate specific pathways in the cancer cells to participate in chemotherapy response. Accordingly, the NET-DNA complex has been shown to induce the cGAS-STING pathway in myeloid cells (19), and one could hypothesize that activation of this pathway in cancer cells could also contribute to tumor suppression.

NETs have demonstrated the ability to induce tissue damage, prompting the question of whether NET-associated CG and subsequent apoptosis contribute to this phenomenon in noncancerous cells. In cancer, chemotherapy is associated with complications like kidney damage and peripheral neuropathy, where NETs play a crucial role (8, 20). Although targeting NETs has been effective in preclinical models to alleviate these complications, it’s essential to consider that anti-NET strategies might simultaneously hinder chemotherapy response, particularly in patients with *PI3KCA*-mutated CRC, as exposed here by Li, et al. (4)

While chemotherapy is conventionally associated with neutropenia, the preclinical models in Li, et al. (4) did not exhibit neutropenia. Instead, chemotherapy resulted in a substantial recruitment of neutrophils in the tumor tissue, a phenomenon observed by other research groups (7, 8). This outcome implies that the recruitment of neutrophils to tumor tissues may contribute to what is traditionally labeled as neutropenia in the bloodstream. Consequently, evaluating whether blood neutropenia in patients correlates with heightened neutrophil recruitment in tumor tissue becomes a critical consideration.

The absence of objective responses in patients treated with the combination drug prompts crucial questions. Despite the positive link between increased NET levels and extended progression-free survival in posttreatment tumor biopsies, understanding the reasons behind this lack of objective responses is essential. The complexities involved in translating promising preclinical results into successful clinical outcomes highlight the need to consider factors such as patient heterogeneity, tumor-specific characteristics, and sample sizes. Altogether, this elegant work by Li, et al. (4) not only elucidates the intricate interplay between NETs and *PI3KCA*-mutated CRC but also highlights the context-dependent nature of these interactions in shaping the response to chemotherapy.

## Version 1 03/01/2024

Electronic publication

## References

[1] Jaillon S (2020). Neutrophil diversity and plasticity in tumour progression and therapy. Nat Rev Cancer.

[2] Galdiero MR (2016). Occurrence and significance of tumor-associated neutrophils in patients with colorectal cancer. Int J Cancer.

[3] Posabella A (2020). High density of CD66b in primary high-grade ovarian cancer independently predicts response to chemotherapy. J Cancer Res Clin Oncol.

[4] Li Y (2024). Neutrophil Extracellular Traps induced by chemotherapy inhibit tumor growth in vivo. J Clin Invest.

[5] Hao Y (2016). Oncogenic PIK3CA mutations reprogram glutamine metabolism in colorectal cancer. Nat Commun.

[6] Zhao Y (2020). 5-fluorouracil enhances the antitumor activity of the glutaminase inhibitor CB-839 against PIK3CA-mutant colorectal cancers. Cancer Res.

[7] Shahzad MH (2022). Neutrophil extracellular traps in cancer therapy resistance. Cancers (Basel).

[8] Mousset A (2023). Neutrophil extracellular traps formed during chemotherapy confer treatment resistance via TGF-beta activation. Cancer Cell.

[9] Shinde-Jadhav S (2021). Role of neutrophil extracellular traps in radiation resistance of invasive bladder cancer. Nat Commun.

[10] Tamura K (2022). Neutrophil extracellular traps (NETs) reduce the diffusion of doxorubicin which may attenuate its ability to induce apoptosis of ovarian cancer cells. Heliyon.

[11] Rot A (1991). Chemotactic potency of recombinant human neutrophil attractant/activation protein-1 (interleukin-8) for polymorphonuclear leukocytes of different species. Cytokine.

[12] Albrengues J (2018). Neutrophil extracellular traps produced during inflammation awaken dormant cancer cells in mice. Science.

[13] (2023). NETworking with cancer: The bidirectional interplay between cancer and neutrophil extracellular traps. Cancer Cell.

[14] Sionov RV (2019). Neutrophil cathepsin G and tumor cell RAGE facilitate neutrophil anti-tumor cytotoxicity. Oncoimmunology.

[15] Nomura M (2003). 14-3-3 Interacts directly with and negatively regulates pro-apoptotic Bax. J Biol Chem.

[16] Cools-Lartigue J (2014). Neutrophil extracellular traps in cancer progression. Cell Mol Life Sci.

[17] Yang L (2020). DNA of neutrophil extracellular traps promotes cancer metastasis via CCDC25. Nature.

[18] Teijeira A (2020). CXCR1 and CXCR2 chemokine receptor agonists produced by tumors induce neutrophil extracellular traps that interfere with immune cytotoxicity. Immunity.

[19] Apel F (2021). The cytosolic DNA sensor cGAS recognizes neutrophil extracellular traps. Sci Signal.

[20] Wang CY (2023). Neutrophil extracellular traps as a unique target in the treatment of chemotherapy-induced peripheral neuropathy. EBioMedicine.

